# Encapsulation of Nicardipine Hydrochloride and Release from Biodegradable Poly(D,L-lactic-co-glycolic acid) Microparticles by Double Emulsion Process: Effect of Emulsion Stability and Different Parameters on Drug Entrapment

**DOI:** 10.1155/2017/1743765

**Published:** 2017-11-08

**Authors:** Nopparuj Soomherun, Narumol Kreua-ongarjnukool, Sorayouth Chumnanvej, Saowapa Thumsing

**Affiliations:** ^1^Department of Industrial Chemistry, Faculty of Applied Science, King Mongkut's University of Technology North Bangkok, Bangkok, Thailand; ^2^Neurosurgery Unit, Surgery Department, Faculty of Medicine Ramathibodi Hospital, Mahidol University, Bangkok, Thailand

## Abstract

Poly(D,L-lactic-co-glycolic acid) (PLGA) is an important material used in drug delivery when controlled release is required. The purpose of this research is to design and characterize PLGA microparticles (PLGA MPs) implants for the controlled release of nicardipine hydrochloride (NCH)* in vitro*. This study used the water-in-oil-in-water (w_1_/o/w_2_) double emulsion and solvent diffusion/evaporation approach to prepare PLGA MPs. Optimal processing conditions were found, such as polymer content, surfactant type, stabilizer concentration, inner and outer aqueous phase volumes, and stirring speed. The PLGA MPs for use as nicardipine hydrochloride (NCH) loading and release had spherical morphology, and the average diameter was smaller than 5.20 ± 0.25 *μ*m. The release kinetics were modeled to elucidate the possible mechanism of drug release.* In vitro *release studies indicated that the NCH release rate is slow and continuous. PLGA MPs are an interesting alternative drug delivery system, especially for use with NCH for biomedical applications.

## 1. Introduction

**Figure 1:**
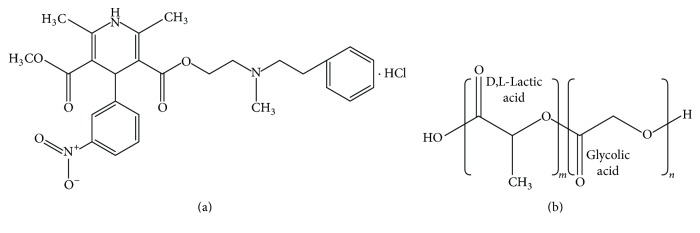
Chemical structure of nicardipine hydrochloride (a) and poly(D,L-lactic-co-glycolic acid) (b).

Currently, in general medical practice, the use of nicardipine hydrochloride (NCH) for the long-term treatment of hypertension (high blood pressure) is common. NCH prevents calcium ion (Ca^2+^) entry into vascular smooth and cardiac muscle through calcium channels [1]. Vasoconstriction, or a blood vessel constriction, is dependent on Ca^2+^, which increases blood pressure. The inhibition of Ca^2+^ movement can be used to treat high blood pressure, angina, and subarachnoid haemorrhage in patients with hypertension [2]. NCH is part of a class of calcium channel blockers and is a hydrophilic drug, as shown in Figure 1(a). As a result, it has a short half-life and has degradable ester linkages in its structure [3, 4]. Therefore, a patient may need to take medication frequently, which may result in toxic effects.

To solve the above problem, researchers have attempted to find a biomaterial for controlled NCH delivery. Biopolymers are biomaterials that can reduce side effects and promote continuous therapeutic medication levels for extended periods of time [5]. Among all biopolymers, poly(D,L-lactic-co-glycolic acid) (PLGA) has shown immense potential as a good drug delivery material. Chemically, PLGA is a copolymer of poly(D,L-lactic acid) (PLA) and poly(glycolic acid) (PGA), as shown in Figure 1(b). As a result of this structure, PLGA is highly biocompatible and biodegradable [6]. PLGA degrades in the body by hydrolysis of the backbone ester linkages into oligomers and finally into nontoxic monomeric compounds. Above all, this material has been approved by the FDA for biomedical applications [7].

In addition, Laura C. et al. modeled the release profiles of risperidone loaded PLGA microparticles (PLGA MPs)* in vitro *and* in vivo *in rats, using varying ratios of the D,L-lactic acid and glycolic acid (50 : 50, 65 : 35, 75 : 25, and 85 : 15). The PLGAs had typical biphasic release profiles extending over 20, 40, 55, and 90 days, respectively [8]. The profiles also showed continued release with increasing D,L-lactic acid. The presence of the methyl side groups in D,L-lactic acid makes it more hydrophobic than glycolic acid; therefore, PLGA is less hydrophilic, absorbs less water, and subsequently degrades more slowly, as PLGA degrades by hydrolysis of its ester linkages in the presence of water [7, 9]. Usually, PLGA is used to prepare capsules for controlling the release of a substance.

The majority of research based on this technology has been focused on a targeted drug delivery system with polymer encapsulation. The polymer encapsulation enhances the specific activity of the main drug, leading to an improvement in pharmacokinetics, modifications to the toxicities associated with a particular drug, protection of the drug from deactivation, and preservation of its activity during transport to the target organ [10, 11]. Current encapsulation techniques include various methods, such as coacervation, spray drying, ionic gelation, and emulsion, each of which obtains a different particle size [12].

The emulsion technique is the best suited to encapsulate hydrophilic and hydrophobic drugs and to improve drug bioavailability, and the particles are highly stable and can be administered in many ways, such as via the gastrointestinal system, the eyes, the skin, or the nose or even via injection into a vein. This is particularly interesting for applications in the pharmaceutical, cosmetic, and food industries [13]. The water-in-oil-in-water (w_1_/o/w_2_) double emulsion is a technique used to prepare PLGA MPs with an encapsulated hydrophilic drug distributed in the PLGA. Drugs formulated in such polymeric devices are released by either Fickian or non-Fickian diffusion through the PLGA barrier and by the erosion of the PLGA material. In addition to their biocompatibility, drug compatibility, suitable biodegradation kinetics, and mechanical properties, PLGA MPs can be easily processed and prepared in various sizes [14]. This is particularly interesting for pharmaceutical and biomedical applications.

With the above motivations, the hypothesis in this research was that these PLGA MPs can effect controlled drug release. For the drug delivery system to be successful, the stable encapsulation of NCH is necessary. PLGA MPs were prepared with the ratio of D,L-lactic acid to glycolic acid at 50 : 50 because this research focuses on controlling NCH release over approximately 15 days [8]. The main goal of the research is to study for the first time the influence of various parameters on the PLGA MPs. This research used the w_1_/o/w_2_ double emulsion and solvent diffusion evaporation approach method to prepare PLGA MPs. PLGA MPs were prepared to find the optimum processing parameters [7], such as polymer content, surfactant type, stabilizer concentration, inner and outer aqueous phase volumes, and speed of stirring for the emulsification process. Finally, the PLGA MPs were compared in terms of size, polydispersity, morphology, encapsulation efficiency, and NCH release.

## 2. Materials and Methods

### 2.1. Materials

Acid-terminated poly(D,L-lactic-co-glycolic acid) (PLGA) with an average molecular weight of 24,000–38,000 Da and a copolymer ratio of D,L-lactide to glycolide at 50 : 50 was purchased from Sigma-Aldrich (USA) to be used as the wall material for microparticles. Sorbitan monooleate (Span 80, Fluka, Switzerland) and poly(vinyl alcohol) (PVA, Mw. ~31,000 Da, Sigma-Aldrich, USA) are often used in foods and cosmetics and were used here as stabilizers. Nicardipine hydrochloride (NCH, LR IMPERIAL, Philippines) and normal saline solution (NSS, Thai-Otsuka, Thailand) were obtained as from Medicine Ramathibodi Hospital, Mahidol University, Thailand. Dichloromethane and acetone were purchased from Lab-Scan (Thailand). All chemical agents were of analytical grade and used without further purification.

### 2.2. Preparation of the PLGA MPs

An appropriate amount of PLGA was dissolved in DCM (15.0 mL) and acetone [15] and mixed until a clear solution was formed. Then, the inner aqueous phase (w_1_) solution, consisting of 3% v/v NCH in NSS, was added to the PLGA organic solution, which was subsequently added to the surfactant. This mixture was homogenized (EL Dorado Labtech) for the 1st emulsion thoroughly in an ice bath, forming the w_1_/o single emulsion.

**Figure 2:**
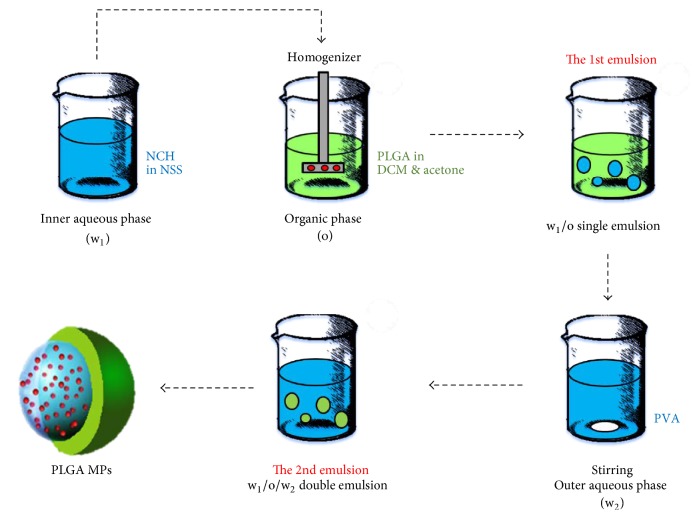
Schematic illustration of the process of forming PLGA MPs via w_1_/o/w_2_ double emulsion.

Next, the w_1_/o single emulsion was added to an outer aqueous (w_2_) solution containing PVA as a stabilizer. For phase separation of PLGA, this was stirred with a magnetic stirrer for the 2nd emulsion, using a specific PLGA amount, w_1_ volume, acetone volume, PVA concentration, and w_2_ volume and speed for the 1st emulsion, as listed in Table 1. The system was thermally maintained in an ice bath to achieve the w_1_/o/w_2_ double emulsion. After that, DCM and acetone were allowed to evaporate. The resulting PLGA MPs were collected by centrifugation, washed three times with DI water, and finally resuspended in 10 mL of phosphate-buffered saline (PBS). The schematic of the setup in this experiment is shown in Figure 2.

### 2.3. Emulsion Stability Measurements

#### 2.3.1. Preparation of Emulsion

The selection of the surfactant is crucial in the formation of emulsion and its long-term stability. First, 2.0 mL of NSS was added to the PLGA solution phase consisting of 100.0 mg of PLGA in 15.0 mL of DCM and 0.5 mL of acetone. Then, 10.0 mg/mL of nonionic surfactants was added to the PLGA solution, and this mixture was homogenized thoroughly at 8,000 rpm. The solution was then allowed to mix in the same manner as the w_1_/o single emulsion. The choice of nonionic surfactant was intended to represent a wide range of hydrophilic-lipophilic balance (HLB) values, as Span 85, Span 80, Tween 80, and PVA are known to have HLB values of 1.8, 4.3, 15.0, and 18.0, respectively [16].

#### 2.3.2. Bottle Test

The stability of the w_1_/o emulsions as a function of the 10.0 mg/mL of nonionic surfactant (Span 85, Span 80, Tween 80, and PVA) was investigated using a bottle test by observation of the phase separation of samples over time. Freshly prepared emulsions were transferred into 10.0 mL graduated glass bottles sealed with a plastic cap and stored for 480 min at room temperature. The phase separation of the emulsions was visually monitored at regular time intervals. Nonseparated phases were observed for all emulsions after 480 min. The percentage of each phase volume in relation to the total volume was calculated. Analyses were performed in triplicate (*n* = 3) [17].

#### 2.3.3. Emulsion Stability Index (ESI)

To evaluate the emulsion stability, the extent of emulsion separation by gravity was assessed by ESI. For this test, 1.0 mL of the freshly prepared, diluted emulsion was transferred to a 20.0 mL test tube and capped to prevent evaporation; tubes were stored at room temperature for 480 min. The absorbance of the diluted emulsions was measured with a UV-Visible spectrophotometer (GENESYS 20 UV-VIS, Thermo Fisher Scientific, USA) at 500 nm in 1 cm path length cuvettes. The percentage of ESI can be identified as the following equation [18]:

(1) $$\begin{array}{c} \%\;\;ESI=\left(\;\frac{2.303A\Delta t}{\Delta T}\;\right), \end{array}$$

where *T* is turbidity at 0 min, *A* is absorbance at 500 nm, Δ*T* is change in turbidity over a 480 min period, and Δ*t* is time interval (480 min).

### 2.4. Measurement of Physicochemical Properties of PLGA MPs

#### 2.4.1. Critical Micelle Concentration (CMC)

Using nonionic surfactants that were found to result in a highly stable emulsion, this research studied the surfactant concentration. The surfactant stock solution was diluted (in the range of 1.0–100.0 mg/mL). Then, the molar conductivity of different concentrations of the surfactant stock solution was determined by electrical conductometry (EUTECH Instruments con 510) at room temperature (25 ± 1°C). Analyses were performed in triplicate (*n* = 3) [19].

#### 2.4.2. Swelling of the PLGA MPs

The swelling behavior of the PLGA MPs that had been prepared with different amounts of PLGA was investigated after incubation at 37°C in 10 mL of 10 mM PBS. After 24 h, the size of the swollen PLGA MPs was analyzed according to the procedure described in the previous subsection. For each sample type, at least 100 microspheres were analyzed, and their swelling behavior was quantified using the following equation [20]:

(2) $$\begin{array}{c} Swelling\;\;ratio=\left(\;\frac{D_{swell}}{D_{dry}}\;\right)\times 100, \end{array}$$

where *D*_swell_ and *D*_dry_ represent the size of the PLGA MPs after and before the incubation, respectively.

#### 2.4.3. Characterization of the PLGA MPs

A scanning electron microscope (SEM, JEOL JSM 6400) was used to obtain electron microscopic images of the PLGA MPs. The PLGA MPs were mounted on double-sided conductive tape attached to the SEM specimen holders. The PLGA MPs were then sputtered with a layer of gold by spraying them with gold vapor for 20 minutes under an argon atmosphere. The PLGA MPs were observed by SEM with an accelerating voltage of 15 kV and under high vacuum. In this work, the diameter sizes for nonstable dispersion were measured from optical microscope (DM 4000 M) by the SemAfore 5.21 software (*n* = 200).

### 2.5. Encapsulation Efficiency (% EE) of NCH in the PLGA MPs

PLGA MPs encapsulating NCH (NCH/PLGA MPs) were prepared by the w_1_/o/w_2_ double emulsion technique. NCH was dissolved in the NSS phase (3.0% v/v). This NCH solution was added to the solution of PLGA in DCM (15.0 mL) and acetone, into which was subsequently added 30.0 mg/mL of Span 80. This mixture was homogenized thoroughly in an ice bath to yield the w_1_/o single emulsion. Next, the w_1_/o single emulsion was added to an aqueous PVA solution and further stirred, with a specific PLGA amount, w_1_ volume, acetone volume, PVA concentration, and w_2_ volume and speed, as listed in Table 1. The amount of NCH encapsulated in PLGA MPs was measured by using a UV-Visible spectrophotometer at a wavelength of 240 nm. The % EE was calculated using the following equation:

(3) $$\begin{array}{c} \%\;\;EE=100-\left(\;\frac{B}{A}\times 100\;\right)-C, \end{array}$$

where *A* was the total amount of NCH, *B* was the unencapsulated NCH in PLGA MPs, and *C* was the NCH adsorbed in w_2_.

### 2.6. Effect of the Stability of the NCH Entrapment in PLGA MPs

The optimum of parameters indicated in Table 1 was used to prepare the PLGA MPs. A set of experiments was performed to study the effect of two different temperatures, 4 and 37°C, in PBS solution (pH 7.4). The NCH remaining was determined using a UV-Visible spectrophotometer at a wavelength of 240 nm.

### 2.7. *In Vitro* Drug Release Profiles

The* in vitro *release kinetics of the PLGA MPs were investigated in PBS solution (pH 7.4) at 37°C. First, this research prepared a 3.0% v/v solution of NCH in 1.0 mL of NSS loaded into 50.0 mg of PLGA in DCM (15.0 mL) and acetone (0.5 mL). The Span 80 concentration was 30.0 mg/mL, and a stirring speed of 8,000 rpm was used to yield the w_1_/o single emulsion. The w_1_/o single emulsion was then added to a 20 mL solution of 5.0% w/v of PVA and further stirred. The system was thermally maintained in an ice bath to achieve the w_1_/o/w_2_ double emulsion. Finally, the preparation of PLGA MPs was suspended in a 50 mL test tube containing 20 mL of PBS solution. The sample tubes were then placed in an incubator and shaken horizontally at 70 rpm. At predetermined times following the beginning of the incubation, the PLGA MPs were retrieved from the solution by centrifugation at 1,000 rpm. The supernatant release medium was removed and replaced with fresh PBS solution each day, and the NCH concentration in the supernatant was determined using a UV-Visible spectrophotometer at a wavelength of 240 nm.

### 2.8. Statistical Analysis

All of results in this research are expressed as the mean ± SD. Significant differences between groups were tested using randomized complete block design (RCBD), followed by a one-way analysis of variance (ANOVA) in SPSS (SPSS, USA). The differences were considered statistically significant when *P* ≤ 0.05.

## 3. Results and Discussion

The purpose of this work was to study preparations of NCH/PLGA MPs by the w_1_/o/w_2_ double emulsion method to achieve release over longer times than NCH without PLGA MPs. The experimental portion is divided into three parts. The first part was a study of parameters that affect emulsion stability. The second part was a study of the parameters affecting the w_1_/o/w_2_ double emulsion method used to prepare the NCH/PLGA MPs, which are shown in Table 1. After the PLGA MPs were prepared, drug encapsulation efficiency and release* in vitro *were studied.

### 3.1. Effects on Emulsion Stability

#### 3.1.1. Effect of Surfactant Type

**Figure 3:**
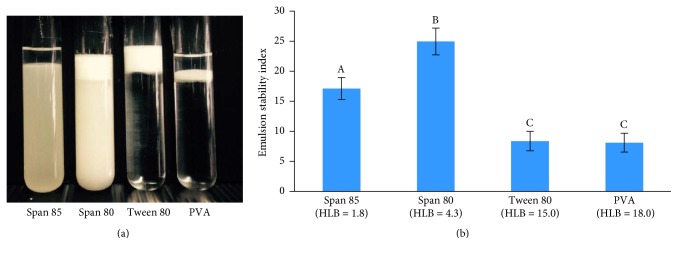
Digital photographs (a) and emulsion stability index (b) of the w_1_/o single emulsion with the addition of surfactants, taken 480 min after preparation (A, B, and C are significantly different at the *P* ≤ 0.05 level, with comparisons among the type of surfactants in each group).

**Figure 4:**
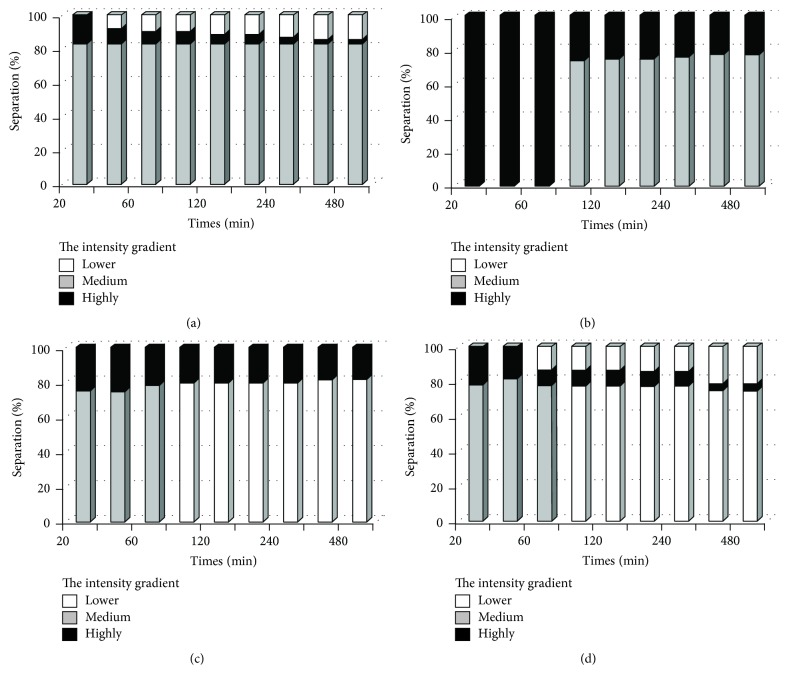
Bottle test results demonstrating phase separation of Span 85 (a), Span 80 (b), Tween 80 (c), and PVA (d).

The surfactant type has an effect on emulsion stability. In this work, this research characterized Span 85, Span 80, Tween 80, and PVA. The results shown in Figure 3(a) imply that all the surfactants can be used to prepare the w_1_/o single emulsion, but emulsion stability was affected by the type of surfactant. For the Span 80 stabilized emulsions, a visual observation of the emulsion indicates that phase separation occurs after 90 min. On the other hand, Span 85, Tween 80, and PVA showed phase separation of the emulsion after 20 min, which appears as the transparent phase shown in Figure 4. This process results from external forces, primarily gravity. When such forces exceed the thermal motion of the droplets (Brownian motion), a concentration gradient builds up in the system with the larger droplets moving faster to the top or the bottom of the container. Emulsion stability refers to the ability of emulsion to resist changes to its concentration gradient over time. Emulsion stability is expressed as ESI [18], shown in Figure 3(b). The stability of emulsions made from Span 80 was significantly higher than that of emulsions prepared from another surfactant type (*P* ≤ 0.05).

The selection of different surfactants in the preparation of either o/w or w/o emulsions is often still made on an empirical basis. The HLB is a measure of the percentage of hydrophilic to lipophilic groups in the surfactant molecules, as described by Griffin [16]. The Span 80 (HLB = 4.3) and Span 85 (HLB = 1.8) structures contain 1 and 3 fatty acid groups, respectively. The HLB value decreases with increasing fatty acid content. Thus, surfactants with HLB < 7 tend to form w/o emulsions. Moreover, Tween 80 (HLB = 15.0) and PVA (HLB = 18.0) are hydrophilic in nature. Their advantage, with HLBs > 7.0, is their ability to support the formation of o/w emulsions [21]. In this study, we prepared NCH/PLGA MPs by the w_1_/o/w_2_ double emulsion method. In the first stage, this research prepared w_1_/o single emulsions. Taken together, these results suggest that Span 80 was the optimal surfactant for this research.

#### 3.1.2. Effect of Surfactant Concentration

**Figure 5:**
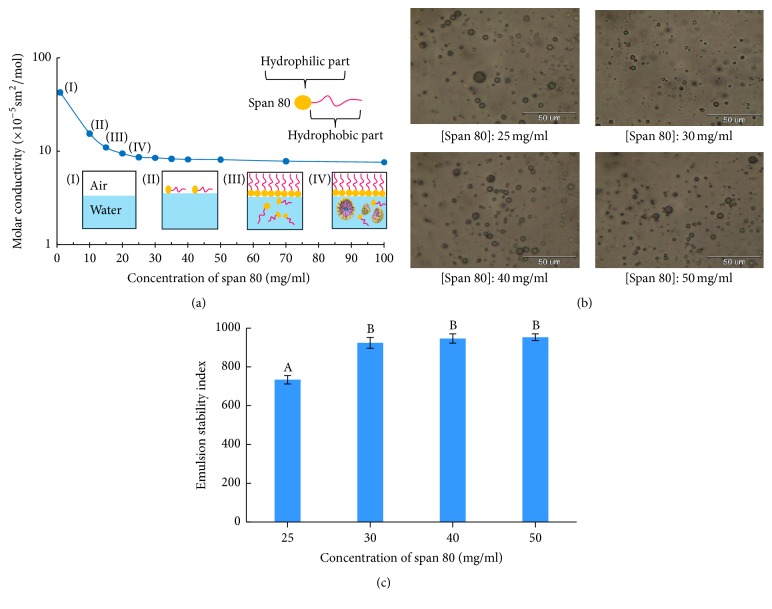
Molar conductivity of Span 80 at different concentrations (a), optical microphotographs following the preparation process of the PLGA MPs (magnification 20x) (b), and emulsion stability index (after 480 min) of the PLGA MPs stabilized by surfactants (c) (A and B are significantly different at the *P* ≤ 0.05 level with comparisons among the PLGA particles in each group).

The results showed that Span 80 conferred the highest emulsion stability in this research and this research studied the effect of different Span 80 concentrations. Then, the CMC was determined by measuring the molar conductivity of different concentrations of Span 80. The CMC is determined with a tensiometer by measuring the surface tension of a concentration series [19]. For Span 80, increasing the concentration decreased the molar conductivity.  Figure 5(a) shows this relationship, and the CMC corresponds to the point on the curve at which a sharp change of slope occurs (15.0 mg/mL). Below the CMC point are few or no micelles, while, beginning at the CMC point, a sharp increase in micelle concentration occurs. Concentrations above 25.0 mg/mL had constant molar conductivity. Therefore, this research studied concentrations in this range (i.e., 25.0 to 50.0 mg/mL). The results shown in Figures 5(b) and 5(c) imply that all concentrations in the selected range can form micelles, but the ESI at 25.0 mg/mL was significantly lower than that of emulsions prepared from another surfactant concentration (*P* ≤ 0.05). Therefore, a Span 80 concentration of 30.0 mg/mL was selected to study the effects of the PLGA amount.

### 3.2. Effects of Different Parameters on Particle Size and NCH Encapsulation Efficiency

#### 3.2.1. Effect of PLGA Amount

**Figure 6:**
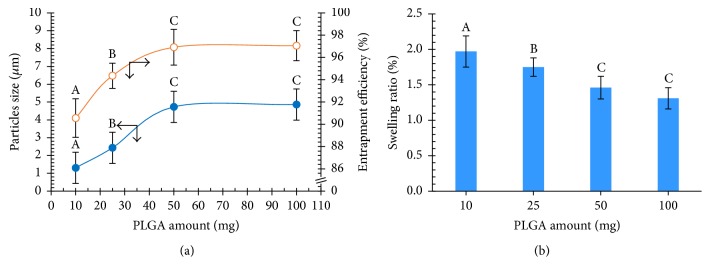
Effects of the PLGA amount on the particle size (blue solid circle) and the entrapment efficiency (orange vacant circle) of the PLGA MPs (a). The ability of PLGA MPs to absorb water and increase in size after swelling in phosphate-buffered saline was studied at 37°C and pH 7.4 for 24 h (A, B, and C are significantly different at the *P* ≤ 0.05 level when comparing in each group).

**Figure 7:**
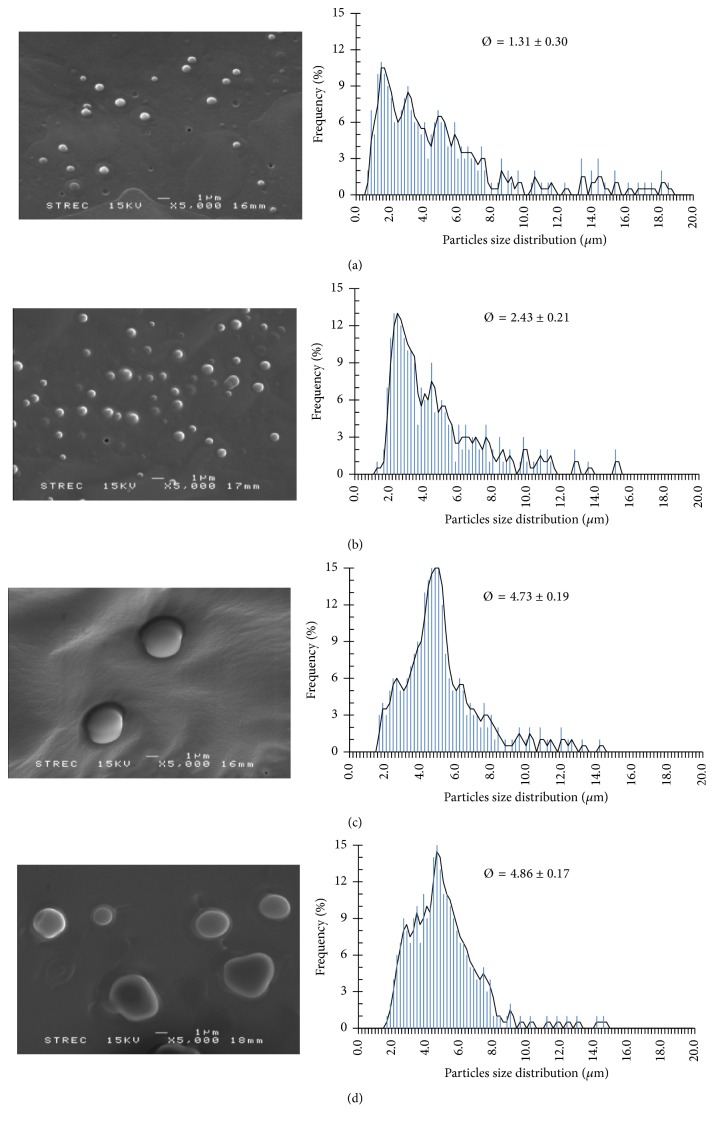
The influence of the PLGA amounts of 10.0 (a), 25.0 (b), 50.0 (c), and 100.0 (d) mg on the morphology and particle size distribution of PLGA MPs from SEM imaging and diameter sizes for nonstable dispersion distribution diagram (magnification 5,000x).

Microparticles of PLGA were prepared to fabricate a biodegradable polymeric carrier for NCH by the w_1_/o/w_2_ double emulsion method. The PLGA amount is an important factor influencing the properties of the particles, such as the PLGA MPs size and encapsulation efficacy. In this work, PLGA was used as the polymer, and the effects of different amounts of PLGA were investigated by keeping all other conditions constant (Table 1). The results shown in Figure 6(a) imply that increasing the PLGA amount led to an increase in the viscosity and chain entanglement, resulting in a significant increase in the size of PLGA MPs. On the other hand, the size distribution of PLGA MPs diminished with increasing PLGA amount, as illustrated in Figure 7. SEM micrographs showed that the PLGA MPs were distributed on the PVA film under all conditions. These issues were resolved when this research studied the effect of w_2_ volume.

The swelling ratio and the swelling behavior of the PLGA MPs made with different amounts of PLGA before and after swelling in PBS were studied at 37°C for 24 h [20]. The diameters of the PLGA MPs from at least 100 freeze-dried and wet samples were measured and calculated, shown in Figure 6(b). The PLGA MPs demonstrated the ability to absorb water and increase in size. The swelling ratios were measured to be 1.2–2.0. Different PLGA amounts did influence the swelling ratio; the swelling ratios decreased significantly when the swelling ratios in each group were compared.

Figure 6(a) illustrates the loading of NCH as a suspension into the PLGA MPs. As the amount of PLGA increased, the encapsulation efficiency increased significantly in each group. However, 50.0 mg PLGA caused the lowest swelling ratios and the highest encapsulation efficiency. An increase in the PLGA amount led to a reduction in the partitioning of the NCH into the w_2_. Indeed, as stated before, PLGA is a copolymer of PLA and PGA. PLA is a more hydrophobic polymer than PGA because of its chemical structure. PGA is a highly crystalline polymer [7]. Therefore, the swelling ratios decrease and the encapsulation efficiency increased when the PLGA amount was increased. These results revealed that, by increasing the amount of PLGA, the particle size and NCH encapsulation efficiency were increased, although the swelling ratio and size were also decreased. A PLGA amount of 50.0 mg was selected as the optimal condition for this research.

#### 3.2.2. Effect of the Inner Aqueous Phase (w_1_) Volume

**Figure 8:**
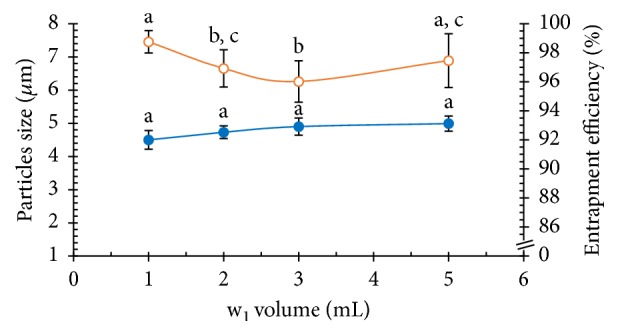
Effects of the w_1_ volume on the particles size (blue solid circle) and the entrapment efficiency (orange vacant circle) of the PLGA MPs (a, b, and c are significantly different at the *P* ≤ 0.05 level when comparing among the w_1_ volumes in each group).

In this work, this research used different volumes of w_1_, including 1, 2, 3, and 5 mL of NSS, to determine the effect on the PLGA MPs. Increasing the volume of the w_1_ does not affect the PLGA MPs size. High amounts of polymer in the solution cause faster coagulation, and chain entanglement occurred when stirring with a high-speed homogenizer when necessary (Table 1). Moreover, the NCH encapsulation efficiency was affected by w_1_ because NCH is a hydrophilic drug whose solubility increases when the w_1_ volume increases. An increase in the volume of the w_1_ led to a decrease in NCH entrapment. The highest NCH encapsulation efficiency was achieved at a w_1_ volume of 1.0 mL. The effect of the w_1_ volume is shown in Figure 8.

#### 3.2.3. Effect of Adding Acetone to the Organic Phase

**Figure 9:**
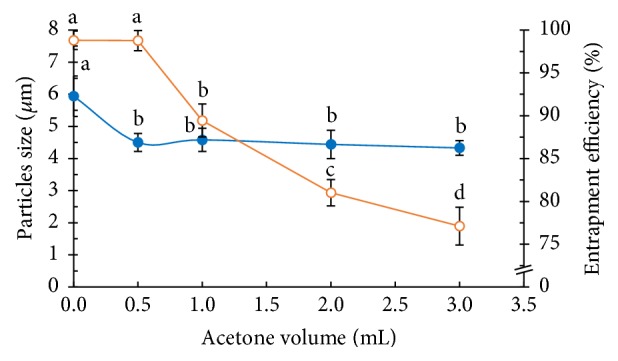
Effect of acetone volume on the particle size (blue solid circle) and the entrapment efficiency (orange vacant circle) of the PLGA MPs (a, b, c, and d are significantly different at the *P* ≤ 0.05 level when comparing among the acetone volumes in each group).

Acetone is a volatile organic cosolvent of PLGA. The PLGA solubility increased upon the addition of a suitable volume of acetone to the organic solution of PLGA [22].  Figure 9 shows that the addition of acetone led to a decrease in the size of the PLGA MPs compared to their size in the absence of acetone. The electrically charged surfaces of the PLGA MPs changed when acetone was added to DCM. Acetone decreased the interfacial tension and increased the movement of the dimensions of the polymer chains in the organic phase, resulting in a decrease in the size of PLGA MPs.

The NCH encapsulation efficiency in the PLGA MPs decreased as increasing amounts of acetone were added. The PLGA solubility increased with increasing acetone volume. Therefore, the NCH diffused easily out of the PLGA MPs. The acetone enhanced the affinity between aqueous and organic phases because of its amphiphilic nature [15]. It promoted the PLGA solubility in the organic phase, with similar solubility parameters (PLGA, DCM, and acetone = 23.8, 20.3, and 19.9 MPa^1/2^, resp.) [23, 24]. An acetone volume of 0.5 mL did not affect the NCH/PLGA MPs, but it made their sizes smaller than those made in the absence of acetone, as shown in Figure 9. Therefore, for this research, 0.5 mL of acetone was selected for further study.

#### 3.2.4. Effects of the PVA Concentration in the Outer Aqueous Phase (w_2_)

**Figure 10:**
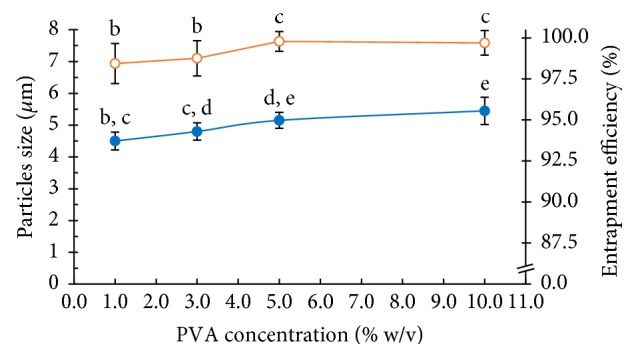
Effect of PVA concentration on the particle size (blue solid circle) and the entrapment efficiency (orange vacant circle) of the PLGA MPs (a, b, c, and d are significantly different at the *P* ≤ 0.05 level when comparing among the PVA concentrations in each group).

The addition of a suitable concentration of PVA plays a key role in the formation of PLGA MPs by w_1_/o/w_2_ double emulsion. Normally, PVA is a biocompatible polymer which has shown a capability to improve pH consistency and lifelong temperature, so PVA is used for the highest stabilizer of PLGA MPs. The properties of PVA can promote suitable mediator for the fabrication of PLGA MPs using in medical applications [25]. The concentration of PVA as a stabilizer affects the size of the PLGA MPs and the NCH encapsulation efficiency, which is illustrated in Figure 10. In the 1.0% w/v of PVA, the encapsulation of NCH in PLGA MPs was very low, and the PLGA MP size was larger than 4.51 *μ*m. Thus, the increasing concentrations of PVA drastically improved the NCH loading and the PLGA MP size. The largest PLGA MP size was that observed in the absence of PVA. Because the PVA is a high molecular weight polymer, the increased concentration of PVA led to an increase in the viscosity of and chain entanglements in w_2_. The small size of the PLGA MPs prepared was achieved with a PVA concentration of 1.0% w/v, but 5.0% w/v of PVA prepared PLGA MPs with high NCH loading. The focus of this research is to prepare PLGA MPs with high NCH entrapment. This research selected a PVA concentration of 5.0% w/v because it resulted in the highest NCH entrapment and a PLGA MP size smaller than the size of red blood cells (~10 *μ*m) [26].

#### 3.2.5. Effect of the Outer Aqueous Phase (w_2_) Volume

**Figure 11:**
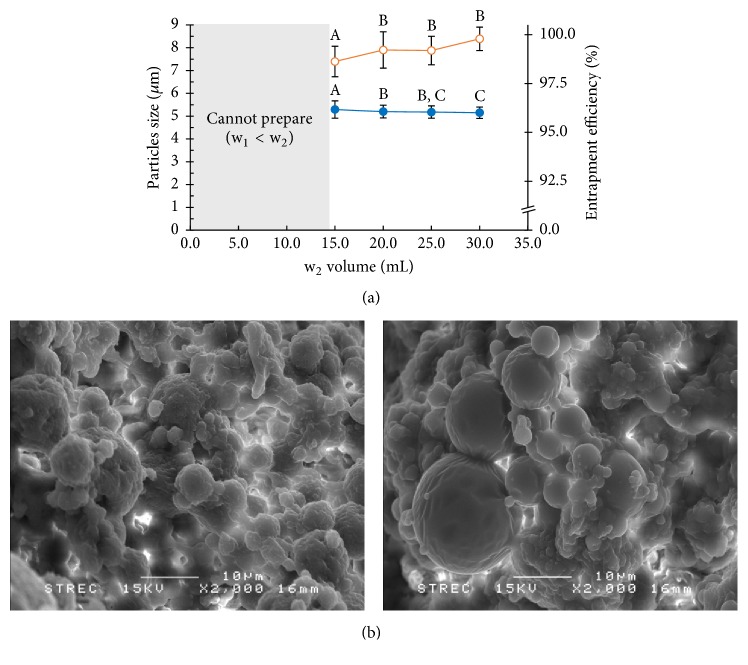
Effects of w_2_ volume on the particle size (blue solid circle) and the entrapment efficiency (orange vacant circle) of the PLGA MPs (a) and SEM image of PLGA MPs fabricated with 15.0 and 20.0 mL of w_2_ volume (b); the scale bar represents 10 *μ*m. (A, B, and C are significantly different at the *P* ≤ 0.05 level when comparing among the w_2_ volumes in each group).

In this process, the w_1_/o/w_2_ double emulsions are created from a w_1_/o single emulsion distributed in a w_2_. The w_2_ has greater volume than w_1_, which allows the preparation of the w_1_/o/w_2_ double emulsion.  Figure 11(a) shows four samples that were prepared with different volumes of w_2_. Increasing the volume of w_2_ decreased the size of the PLGA MPs. On the other hand, the NCH/PLGA MPs increased as an effect of this, but the volumes used in this study, starting from 20.0 mL, did not affect the NCH entrapment. This may be due to a decrease in the viscosity of the emulsion in higher volumes of w_2_, causing more efficient shearing forces that reduced the PLGA MP size. The effect of this parameter was studied by comparing the SEM photographs of two samples, of which the first used a w_2_ volume of 15.0 mL and the second a volume of 20.0 mL. From the SEM micrographs (Figure 11(b)), it was found that the PLGA MPs prepared by using 15.0 mL of w_2_ did not have smooth surface morphology. On the other hand, the particles prepared with 20.0 mL of w_2_ had regular rounded shapes and a narrow size distribution, resulting in an increase in the NCH entrapment. The issue of the distribution of PLGA MPs on the PVA film, as shown in Figure 7, was addressed by washing the PLGA MPs three times with DI water. Therefore, 20.0 mL of the w_2_ volume was selected as the optimal condition for this research.

#### 3.2.6. Effect of Stirring Speed for the 1st Emulsion

**Figure 12:**
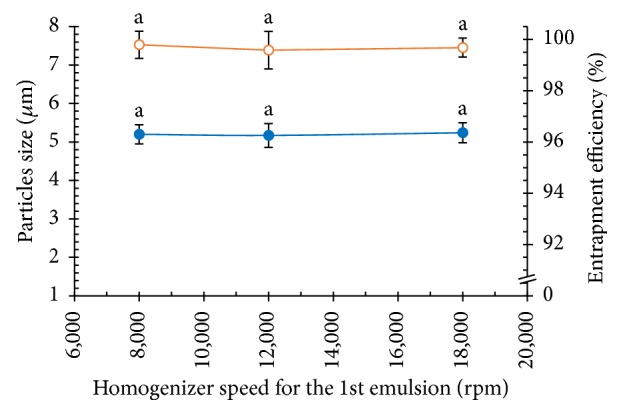
Effects of homogenizer speed for the 1st emulsion on the particle size (blue solid circle) and the entrapment efficiency (orange vacant circle) of the PLGA MPs (a is significantly different at the *P* ≤ 0.05 level when comparing among the homogenizer speeds in each group).

An emulsion is a mixture of two or more immiscible liquids. High energy is applied to the system. In this work, the necessary energy was supplied to the system via high stirring using a homogenizer as shown in Table 1. It is obvious from Figure 12 that changes in the stirring speed for the 1st emulsion during emulsion preparation had no influence on the size of PLGA MPs and the NCH encapsulation efficiency [27]. The effect on both was a slight change with extreme changes in stirring speed. The system agglomerated fast because of the chain entanglement of the PLGA solution.

### 3.3. Effects on the Stability of the Entrapment in PLGA MPs

**Figure 13:**
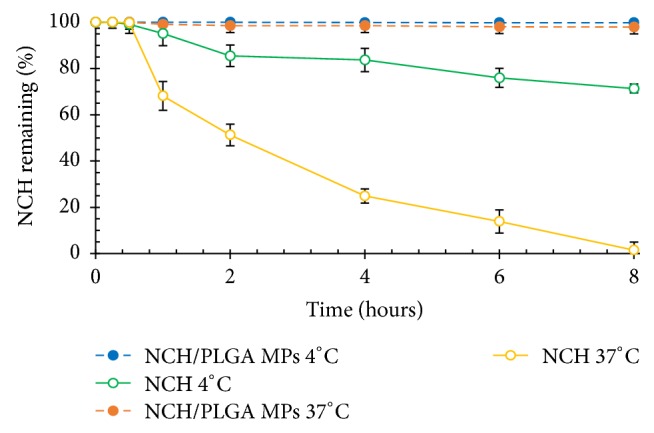
NCH remaining when entrapped in PLGA MPs and in the absence of PLGA MPs at 4 and 37°C in PBS solution (pH 7.4).

In water, NCH degrades by hydrolysis of the ester linkages in the structure [3, 4]. Due to the potential for hydrolysis of NCH, this research believed that PLGA MPs in this research would afford more stability than the absence of PLGA MPs [28]. This investigation was carried out as a demonstration at two different temperatures, that is, 4 and 37°C in PBS solution (pH 7.4). The remnants of NCH decreased at both temperatures, but the NCH/PLGA MPs retained much more NCH alone. The PLGA MPs can preserve the stability of NCH, as shown in Figure 13.

At 4 and 37°C, NCH/PLGA MPs had 99.68% and 97.90% of the remaining NCH, respectively. Therefore, the PLGA MPs are colloids. In general, the disperse phase of a colloid is thermodynamically unstable with respect to the bulk [27]. The increase in temperature had a tendency to cause agglomeration of the PLGA MPs. This phenomenon was responsible for the NCH degradation.

### 3.4. *In Vitro* Drug Release Profiles

**Figure 14:**
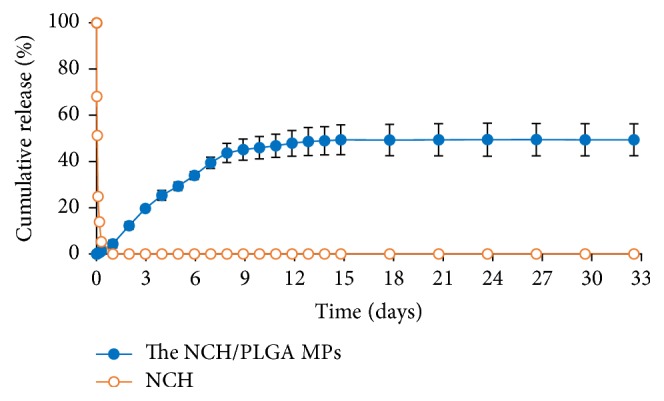
The* in vitro *release profiles of NCH from PLGA MPs incubated in testing medium with 20.0 mL PBS at pH 7.4 and 37°C to mimic the body fluids in normal tissues (*n* = 3).

**Figure 15:**
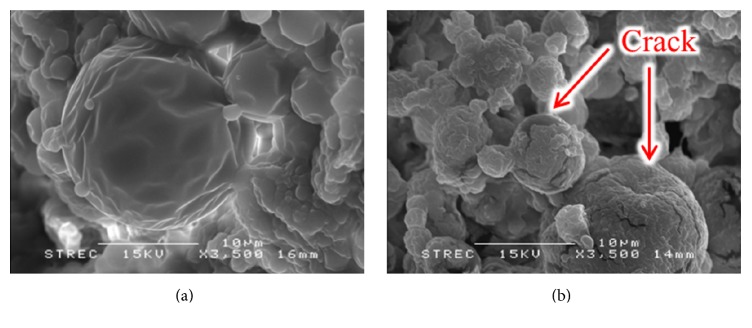
Scanning electron microphotographs illustrating morphology of the PLGA MPs before* in vitro* release (a) and after* in vitro* release (b); (magnification 3,500x).

The aim of this study was to prepare the NCH entrapped in a polymeric system for controlled release as a drug delivery system for vasodilatation agent. This agent is very important for neurosurgeons to prevent cerebral vasospasm after intracranial aneurysmal clipping. The samples were immersed in PBS (pH 7.4) and incubated in a water bath at 37°C with constant shaking at 70 rpm. Whereas* in vitro *release studies on the NCH/PLGA MPs indicated that the NCH is released continuously, NCH release leveled off after the first 33 days.* In vitro *release profiles demonstrated a biphasic modulation. The first phase was characterized by a relatively rapid initial release and followed by a second slower phase. As observed, the* in vitro* release profile of the NCH/PLGA MPs was stopped at 50% (1.5 mg) which is sufficient to sustain an effective drug [29]. Hence, this research has demonstrated the* in vitro* release profile of NCH released from PLGA MPs at destined rate to prolong drug concentration. On the other hand, the NCH* in vitro *release in the absence of PLGA MPs demonstrated NCH degradation over a period of 1 day. NCH has a short half-life [4], as shown in Figure 14; hence NCH was entrapped in PLGA MPs. PLGA MPs have been shown to be excellent delivery systems for controlling the administration of NCH due to their biocompatibility and biodegradability [30]. In Figure 15, the morphology of the NCH/PLGA MPs before and after releasing in PBS solution is studied. In our experiment, the result from Figure 15(a) illustrated that the NCH/PLGA MPs before releasing process provided a sphere shape with smooth surface.  Figure 15(b) showed that it was impossible for drug release for 33 days to see a cracked NCH/PLGA MPs due to PLGA degradation from hydrolysis reaction.

## 4. Conclusions

The optimal processing conditions for the w_1_/o/w_2_ double emulsion method of preparing PLGA MPs include 3.0% v/v of NCH in 1.0 mL of NSS, and this NCH solution is added to organic phase consisting of 50.0 mg of PLGA in DCM (15.0 mL) and acetone (0.5 mL). The surfactant is 30.0 mg/mL of Span 80 with a stirring speed of 8,000 rpm to yield the w_1_/o single emulsion. Next, the w_1_/o single emulsion is added to 20 mL of a 5.0% w/v solution of PVA and further stirred. The system is thermally maintained in an ice bath to form the NCH/PLGA MPs. The average size of the NCH/PLGA MPs was approximately at 5.20 *μ*m, and percentage encapsulation efficiency of NCH in the NCH/PLGA MPs was approximately 99%. The NCH release from PLGA MPs was constant. As a result, this drug delivery system will be an option to demonstrate the choice of treatment for intracranial vasospasm problem.

## Figures and Tables

**Table 1:** Parameters used in different sets of experiments. The “bold” values show the parameters, which were changed in their respective recipes.

Studied parameters	w_1_/o/w_2_ double emulsion's parameter					
	PLGA (mg)	w_1_ (mL)	Acetone (mL)	PVA (% w/v)	w_2_ (mL)	Speed (rpm)
PLGA amount used	10.0	2.0	0.5	3.0	30.0	8,000
	25.0	2.0	0.5	3.0	30.0	8,000
	50.0	2.0	0.5	3.0	30.0	8,000
	100.0	2.0	0.5	3.0	30.0	8,000

w_1_ volume	50.0	1.0	0.5	3.0	30.0	8,000
	50.0	2.0	0.5	3.0	30.0	8,000
	50.0	3.0	0.5	3.0	30.0	8,000
	50.0	5.0	0.5	3.0	30.0	8,000

Acetone volume	50.0	1.0	**Control**	3.0	30.0	8,000
	50.0	1.0	0.5	3.0	30.0	8,000
	50.0	1.0	1.0	3.0	30.0	8,000
	50.0	1.0	2.0	3.0	30.0	8,000
	50.0	1.0	3.0	3.0	30.0	8,000

PVA concentration	50.0	1.0	0.5	1.0	30.0	8,000
	50.0	1.0	0.5	3.0	30.0	8,000
	50.0	1.0	0.5	5.0	30.0	8,000
	50.0	1.0	0.5	10.0	30.0	8,000

w_2_ volume	50.0	1.0	0.5	5.0	15.0	8,000
	50.0	1.0	0.5	5.0	20.0	8,000
	50.0	1.0	0.5	5.0	25.0	8,000
	50.0	1.0	0.5	5.0	30.0	8,000

Stirring speed for the 1st emulsion	50.0	1.0	0.5	5.0	20.0	**8,000**
	50.0	1.0	0.5	5.0	20.0	**12,000**
	50.0	1.0	0.5	5.0	20.0	**18,000**
